# Roles of Proliferation and Angiogenesis in Locally Aggressive Biologic Behavior of Ameloblastoma versus Ameloblastic Fibroma

**DOI:** 10.3390/diagnostics12020392

**Published:** 2022-02-03

**Authors:** Amr Ibrahim, Emad Alqalshy, Ahmed Abdel-Shakour Abdel-Hafiz, Kamal Abd El-Rahman, Magdy Alazzazi

**Affiliations:** 1 Oral and Dental Pathology Department, Faculty of Dental Medicine (Boys-Cairo), AL-Azhar University, Cairo 11651, Egypt; emadalqalshy.209@azhar.edu.eg (E.A.); Dr.ahmedshokry1@gmail.com (A.A.-S.A.-H.); drkamal96@yahoo.com (K.A.E.-R.); 2 Basic Dental Sciences Department, Faculty of Dentistry, Deraya University, New Minya 61768, Egypt; 3 Oral Biology Department, Faculty of Dental Medicine (Boys-Cairo), AL-Azhar University, Cairo 11651, Egypt; magdyelabasiry@gmail.com; 4 College of Dentistry, The Islamic University, Najaf 54001, Iraq

**Keywords:** proliferation, angiogenesis, biologic behavior, ameloblastoma, ameloblastic fibroma

## Abstract

(1) Background: The present study was carried out to evaluate the roles of proliferation and angiogenesis in locally aggressive biologic behavior of ameloblastoma versus ameloblastic fibroma; (2) Methods: 30 formalin-fixed paraffin embedded blocks (15 cases of ameloblastoma and 15 cases of ameloblastic fibroma) were used. To evaluate the proliferation, the tissue sections were stained with an AgNORs stain. CD105 was used as an immunohistochemical marker of angiogenesis. Quantitative evaluations of AgNORs were performed. The mean vascular density was evaluated as a measure for CD105 protein expression by using image analyzer computer system; (3) Results: The mean number of AgNORs dots per nucleus was significantly higher in ameloblastoma as compared to ameloblastic fibroma. Additionally, the protein level of CD105 showed positive expression and wide distribution that the mean vascular density was significantly higher in ameloblastoma as compared to ameloblastic fibroma; (4) Conclusion: Quantitative evaluation of the AgNORs stain and the mean vascular density utilizing CD105 protein expression may reflect a higher proliferative activity and a more locally aggressive biologic behavior of ameloblastoma when compared to ameloblastic fibroma, indicating that other factors may be involved in biologic behavior of ameloblastic fibroma.

## 1. Introduction

Odontogenic tumors are rare lesions that arise from odontogenesis-related cells and tissues, as well as their remains. These tumors are a diverse category of lesions that include hamartomatous lesions as well as benign and malignant neoplasms [1]. The World Health Organization (WHO) classification, which has been refined, categorized the benign odontogenic tumors into those of odontogenic epithelial origin, mixed (Epithelial-Mesenchymal) origin, and mesenchymal origin [1]. Ameloblastoma is a rare, odontogenic tumor derived from odontogenic epithelium. Ameloblastoma represents about 1% of all jaw tumors but is considered to be the second-most common odontogenic tumor, although benign, and is known for its locally invasive behavior with a high risk of recurrence [2]. Ameloblastic fibroma is a rare mixed odontogenic tumor which constitutes 2% of odontogenic tumors. However, some researchers believe that at least some cases of ameloblastic fibroma are hamartomatous and represent the first stage of an odontoma’s development. Nonetheless, the neoplastic origin of ameloblastic fibroma appears to be favoured due to high recurrence potential [3].

Knowledge of the biologic behavior of pathologic entities that affect the oral cavity, such as odontogenic tumors, is crucial for choosing the optimal treatment approach and defining a prognosis for each patient. Proliferative activity is an important predictor of biologic behavior of pathologic condition and a potential guide for therapy. Epithelial proliferations are playing a significant role in the behavior of odontogenic tumors [4].

Nucleolar organizer regions (NORs) are DNA loops that actively transcribe to ribosomal RNA, which then transcribes to ribosomes and, eventually, to protein. The NORs are linked to non-histonic acidic argyrophilic proteins, which can be seen using the AgNORs technique. In hyperplastic and neoplastic conditions, quantitative assessments of NORs can indicate the degree of cell nucleolar activity [5]. Several studies have confirmed the usefulness of AgNORs in reflecting the biologic behavior and cellular proliferative activity of odontogenic tumors [5,6,7].

The connective tissue stroma supports epithelial alterations and expansion through angiogenesis since odontogenic epithelial cells lack a vascular system. In neoplasia, a lack of nutrients and oxygen results in cells’ death due to a lack of neo-vascularization. The existence of myofibroblasts and blood vessels in the connective tissue stroma is critical for neoplastic growth, and angiogenesis is essential for that proliferation. Angiogenesis is a series of events that results in the development of new blood vessels from pre-existing vessels. Proliferation and endothelial cell migration, proteolytic breakdown of extracellular matrix, capillary formation, loop and lumen development, and micro vessel anastomosis are among the mechanisms studied [8]. Immunohistochemical labelling with monoclonal antibodies to endothelial cell antigens can reveal angiogenic vessels. In contrast to regular endothelial cells markers, CD105 is an ideal angiogenesis marker that identify the quality and quantity of newly created capillaries, because it is expressed differently in angiogenesis to in normal endothelial cells (CD31 and CD34) [9,10].

As angiogenesis reflects the growth potential of odontogenic tumors, measuring mean vascular density can help anticipate the biologic behavior of these lesions [10,11,12]. Therefore, the present study was carried out to evaluate the roles of proliferation and angiogenesis in locally aggressive biologic behavior of ameloblastoma versus ameloblastic fibroma.

## 2. Materials and Methods

### 2.1. Samples Selection

A total of 30 formalin-fixed paraffin embedded blocks (15 cases of ameloblastoma and 15 cases of ameloblastic fibroma) were used; they were collected from the archives of the Pathology Department, the National Cancer Institute (Cairo University), Oral & Dental Pathology Departments, Faculty of Dental Medicine, Al-Azhar University and Faculty of Oral & Dental Medicine, Cairo University. Histological diagnosis as well as any relevant clinical data of selected cases were taken from the accompanying clinical sheet. Tissue sections were cut at a thickness of 5 μm and stained with hematoxylin and eosin (H&E) to confirm the diagnosis and AgNORs stain. AgNORs staining was carried out according to the standard staining method [13].

### 2.2. Immunohistochemistry

Immunohistochemistry was used to apply CD 105 antibody to tissue sections in this study. After coating Super-frosted slides with Poly-l-lysine, one section from each block, measuring 3–4 m in thickness, was taken. The CD105 antibody (Dako) was used to react with these sections utilizing the avidin–biotin–peroxidase complex technique. The sections were dried in a hot air oven at 60 °C for 60 min, then dewaxed by immersion in xylene twice for 5 min, 100% and 90% ethanol for 3 min twice each, and washing with distilled water for 2 min twice. Proteinase K was used to retrieve antigen, which was kept at 37 °C for 30 min. Washing with water for 10 min was followed by 3 changes of washing with phosphate buffer saline (PBS) for 2 min each. On the slide, 1 to 2 drops of hydrogen peroxidase block were placed, incubated for 10 min, and then washed in PBS. This was followed by a 2–3 min soak in a buffer bath. Primary antibodies were diluted 1:10 according to the manufacturer’s recommended dilutions (Dako). On the slides, 2 drops of primary antibody were put and incubated for 1 h. This was followed by 30 min of linker solution application and 2–3 min in the buffer bath. After that, the biotinylated secondary antibody reagent (10 min) and streptavidin peroxidase reagent (10 min) were applied, followed by PBS washing and a buffer bath. Finally, a chromogenic substrate solution was applied for 10 min before being washed. Prior to assessment, slides were dehydrated by immersing them in 70%, 90%, and 100% ethanol for 5 min each, followed by immersion in Xylene for 5 min and mounting with DPX [14].

### 2.3. Staining Interpretations

Quantitative analysis of AgNORs was carried out using a standard light microscope [15]. AgNORs stains appeared as distinct intra-nuclear black dots that were manually counted in 100 nuclei at a magnification of 1000× with oil immersion in all tissue sections investigated. Finally, each case’s mean value and standard deviation were calculated.

Under a light microscope, stained sections were interpreted microscopically. Micro vessels were identified as vascular endothelial cells, which were labelled with CD105 and easily identified by brown cytoplasmic staining. Only strong positively stained endothelial cells or cell clusters that were easily differentiated from adjacent micro vessels and other connective tissue elements were regarded as unique countable micro vessels, according to Weidner’s (Weidner et al., 1993) criteria [16]. Ten separate fields with a high number of vessels were randomly chosen under ×100 magnification. At ×400 magnification (about 0.18 mm^2^ field size), micro vasculatures were counted in each of the ten fields. A twin head microscope was used by two operators to evaluate all of the slides at the same time. Each micro vessel had to be agreed upon by both of them before it was counted. The mean vascular density was computed as the average number of vessels per high-power field, and the results were then documented according to the protocol.

### 2.4. Statistical Analysis

Data were represented as mean and standard deviation (SD) values. Student’s *t*-test was used to compare between the studied tissue sections. Spearman’s correlation coefficient was used to determine correlations between the two stains. The significance level was set at *p* ≤ 0.05. Statistical analysis was performed with IBM SPSS Statistics Version 20 for Windows.

## 3. Results

### 3.1. AgNORs Stain Results

**Figure 1:**
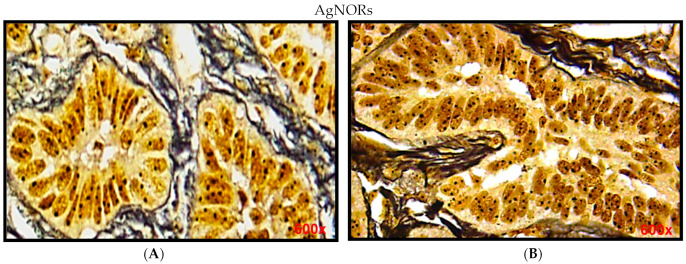
(**A**) Photomicrographs of ameloblastoma showing black dots in the nucleus of peripheral columnar ameloblast-like cells and central stellate shaped cells; (**B**) photomicrographs of ameloblastic fibroma showing black dots in the nuclei of peripheral columnar ameloblast-like cells and central stellate-shaped cells.

Quantitative assessment showed that the mean number of AgNORs dots per nucleus was 1.65 ± 0.24 for ameloblastoma (Table 1), located in the nuclei of peripheral cells (columnar ameloblast-like cells) and central cells (stellate reticulum-like cells) of odontogenic epithelial tumor cells (Figure 1A). Quantitative assessment showed that the mean number of AgNORs dots per nucleus was 1.12 ± 0.21 for ameloblastic fibroma (Table 1), located in nuclei of peripheral cells (columnar ameloblast- like cells) and central cells (stellate reticulum-like cells) of the neoplastic odontogenic epithelial portion (Figure 1B). There were significant differences between the mean numbers of AgNORs dots per nucleus in ameloblastoma when compared with ameloblastic fibroma *p* = 0.03 (Table 1).

### 3.2. CD105 Protein Expression Results

**Figure 2:**
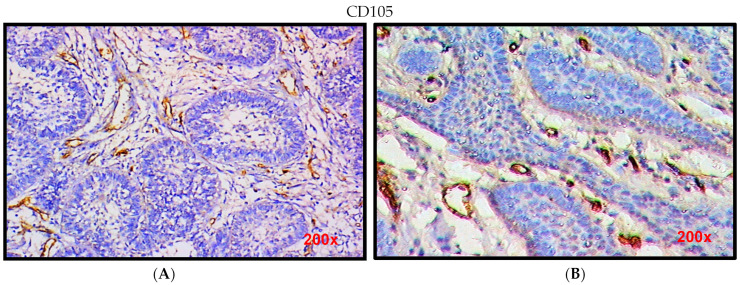
(**A**) Photomicrographs of ameloblastoma showing immunohistochemical staining of micro vessels adjacent to epithelial nests; (**B**) photomicrographs of ameloblastic fibroma showing immunohistochemical staining of micro vessels adjacent to epithelial islands.

Immunohistochemical expression of CD105 in ameloblastoma cases showed positive expression and wide distribution in the endothelial lining of newly formed blood vessels (BVs) (Figure 2A) the mean vascular density was 27.52 ± 7.85 (Table 2) surrounding the odontogenic epithelial tumor cells. Immunohistochemical expression of CD105 in ameloblastic fibroma cases showed positive expression in the endothelial lining of newly formed BVs, the distribution was less prominent (Figure 2B), and the mean vascular density was 18.2 ± 3.57 surrounding the odontogenic epithelial tumor cells. There were significant differences between the mean vascular density in ameloblastoma when compared to ameloblastic fibroma *p* = 0.01 (Table 2).

## 4. Discussion

Odontogenic tumors are a diverse group of lesions with a variety of biologic behaviors. Ameloblastoma is a benign epithelial odontogenic tumor that primarily affects the posterior mandible. Although benign, this tumor has the potential to become aggressive and recur [1]. Ameloblastic fibroma is a mixed odontogenic tumor that frequently affects the posterior mandible. It is a benign, slowly growing, expansile tumor [17]. Some researchers have utilized silver nitrate staining to differentiate the grade of lesions, predict recurrence, and even predict aggressive behavior. It should also be mentioned that the majority of oral pathology research has focused on squamous cell carcinoma [18], and salivary gland tumors [18], with only a few studies focusing on odontogenic lesions. Due to the technique’s simplicity and repeatability, counting is the most extensively utilized approach for testing AgNORs [19]. The roles of AgNORs and the mean vascular density in locally aggressive biologic behavior of ameloblastoma versus ameloblastic fibroma were obtained in this study.

In the present study, quantitative analysis revealed that the mean number of AgNORs dots in ameloblastoma was significantly higher than in ameloblastic fibroma, indicating that ameloblastoma has a higher proliferative activity. These findings are consistent with prior research, which has found that ameloblastoma has a higher AgNORs index than other benign odontogenic lesions [20], and that this index correlates with the growth potentiality of these lesions. In another similar study, AgNORs were used to assess the proliferative potential of dentigerous cysts, keratocystic odontogenic tumors, conventional ameloblastoma, and unicystic ameloblastoma using quantitative and qualitative criteria. When compared to the other tumors in the study, conventional ameloblastoma had the highest mean number of AgNORs [21].

Interactions between the epithelial and mesenchymal components of developing dental tissues influence odontogenesis. As odontogenic tumors emerge from odontogenesis tissue remnants, these interactions have been thought to play a key role in the production of odontogenic tumors. The connective tissue stroma is critical for epithelial tissue preservation, and modest changes in the epithelium are followed by comparable changes in the stroma, such as angiogenesis [9]. Other endothelial markers have shown less selectivity for tumor vasculature than CD105 [22].

The protein level of CD105 in the current study indicated positive expression, wide distribution, and an increase in the mean vascular density, which was significantly higher in ameloblastoma than in ameloblastic fibroma. The findings of the mean vascular density in this study support that the angiogenesis has an important role in tumor progression, invasiveness and the locally invasive biologic behavior of ameloblastoma, which is comparable to the findings of ameloblastic fibroma. These findings are consistent with those of Martano et al. [23]. As a result, it is vital to realize that the epithelium in odontogenic tumors induces angiogenesis in connective tissue, and the formation of new blood vessels around the odontogenic epithelium for nutrients and oxygen facilitates tumor growth. By using Ki-67 and CD34, Liu et al. [24], evaluated the proliferative activity and biologic behavior of ameloblastic fibroma in an 8-year-old male rhesus macaque with unilateral enlargement of the left mandible. Their findings demonstrated that the neoplasm’s epithelial and ectomesenchymal components exhibited modest proliferation.

## 5. Conclusions

Quantitative evaluation of the AgNORs stain (as a tool of proliferation) and immunohistochemical evaluation of the mean vascular density, utilizing CD105 protein expression (as indicator of angiogenesis) may reflect a higher proliferative activity and a more locally aggressive biologic behavior of ameloblastoma when compared to ameloblastic fibroma.

## Figures and Tables

**Table 1:** Comparison between AgNORs stain results in ameloblastoma versus ameloblastic fibroma.

**Tumor Type**	**AgNORs Stain Results**		*p* = 0.03
	**Mean**	**SD**	
Ab	1.65	0.24	
AF	1.12	0.21	

**Table 2:** Comparison between CD105 protein expression results in ameloblastoma versus ameloblastic fibroma.

**Tumor Type**	**CD105 Results**		*p* = 0.01
	**Mean**	**SD**	
Ab	27.52	7.85	
AF	18.2	3.57	

## Data Availability

The data sets used during current study are available from the corresponding author on reasonable request.
